# ANNOUNCEMENTS & RESOURCES

**Published:** 2018-07-31

## Biography traces the steps of a trachoma pioneer

Ophthalmic surgeon Arthur Ferguson MacCallan (1872–1955) worked in Egypt between 1903 and 1923. The MacCallan Classification of Trachoma (now replaced by the WHO grading system) was the first grading system used to standardise the diagnosis of the disease. Grandson Michael MacCallan has now published the second edition of his book about Arthur: *Light out of Deep Darkness. A biography of Arthur Ferguson MacCallan, the trachoma pioneer* (2nd edition).

**Figure:**
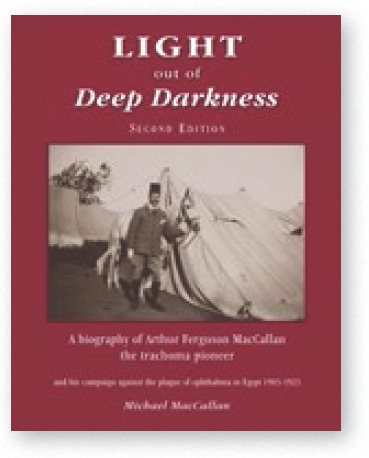


**Reader offer.** *Community Eye Health Journal* readers can order the book for the reduced price of £30 plus postage and packing (usual price £45). Contact The Choir Press at **enquiries@thechoirpress.co.uk** or by telephone on +44 (0) 1452 500 016, quoting reference CEHJ09. Offer valid until 30 September 2018.

## Obituary: Professor Janet Marsden

**Figure:**
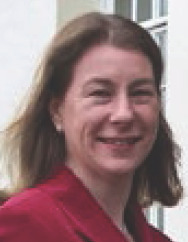


Former *Community Eye Health Journal* Nursing Advisor, Janet Marsden, has passed away on the 31st of May 2018. Janet was Professor of Ophthalmology and Emergency Care at Manchester Metropolitan University and has authored several nursing articles in this journal. She was the editor of *Ophthalmic Care* (Wiley) and later made a significant contribution to the International Centre for Eye Health's Ophthalmic Operating Theatre Practice: a Manual for Lower-resource Settings (**https://www.cehjournal.org/resources/ootp/**). Janet's insight, experience and kindness will be missed very much, and we extend our deepest condolences to her family.

## Courses

### MSc Public Health for Eye Care, London School of Hygiene & Tropical Medicine

Fully funded scholarships are available for Commonwealth country nationals. The course aims to provide eye health professionals with the public health knowledge and skills required to reduce blindness and visual disability.

For more information visit **www.lshtm.ac.uk/study/masters/mscphec.html** or email **romulo.fabunan@lsthm.ac.uk**

### Free online courses

**The ICEH Open Education for eye care programme** offers a series of online courses in key topics in public health eye care. All the courses are free to access and include: Global Blindness, Eliminating Trachoma, Ophthalmic Epidemiology: Basic Principles and Application to Eye Disease.

More free courses coming! Certification also available.

For more information visit **http://iceh.lshtm.ac.uk/oer/**

## Subscriptions

Contact Anita Shah

**admin@cehjournal.org**

### Subscribe to our mailing list

**web@cehjournal.org** or visit **www.cehjournal.org/subscribe**

### Visit us online

**www.cehjournal.org**

**www.facebook.com/CEHJournal**

**https://twitter.com/CEHJournal**

